# Microfabrication of re-entrant surface with hydrophobicity/oleophobicity for liquid foods

**DOI:** 10.1038/s41598-020-59149-2

**Published:** 2020-02-10

**Authors:** Masaki Yamaguchi

**Affiliations:** Graduate School of Medicine, Science & Technology, Shinshu University, 3-15-1 Ueda, Nagano, 386-8567 Japan

**Keywords:** Biomedical engineering, Mechanical engineering

## Abstract

Re-entrant texturing may potentially improve the hydrophobicity and oleophobicity of a surface. The food industry requires a microfabrication method to keep surfaces clean without leaving a packaging residue for applications such as food bottles, food containers, and preservation bags. The goal of this study is thus to establish a microfabrication method for re-entrant texturing with spherical curvature to produce hydrophobic/oleophobic surfaces for liquid foods, such as soy sauce and canola oil. Samples with a spherical curvature are created from an ultra-violet-cure (UV-cure) resin and poly (tetrafluoroethylene) (PTFE) microbeads with diameters between 2.26 to 1,353 microns by spin coating on a glass substrate. The resin thickness, the mass and diameter of the microbeads, and the spin coater rotation speed are used as the microfabrication parameters. A side view of samples showing the spherical curvature reveals that a re-entrant texture indeed forms. Distilled water, soy sauce, and canola oil are dropped softly onto the re-entrant surface, however, the droplets cannot be placed stably. For appropriate microbead diameters, the apparent contact angles of soy sauce and canola oil showed 130.2 and 119.4 degrees, respectively. This facile fabrication method for re-entrant surfaces could prove useful for generating hydrophobic/oleophobic surfaces for Newtonian liquid foods.

## Introduction

Superhydrophobic surfaces exhibit apparent contact angles with water (surface tension γ_LV_ = 72.1 mN/m) that exceed 150°, and low contact angle hysteresis is controlled by two factors: the chemical composition and the surface roughness. Barhlott presented technical applications for such features by using micro- and nano-sized particles for applications, including building materials and food containers^1^. However, superhydrophobic surfaces with contact angles exceeding 150° are extremely rare in nature. Tuteja *et al*. reported that a third factor, the re-entrant surface curvature, can be used to design surfaces with extreme resistance to wetting for a number of liquids with low surface tension^2,3^. For re-entrant curved surfaces (re-entrant texture), the net traction on the liquid–vapor interface is directed upwards, thereby supporting the formation of a composite interface^4^. Additionally, four design criteria have been proposed to form an air-entrapped Cassie–Baxter state from the re-entrant texturing considering the pressure balance of liquids, such as Laplace pressure, gravity, surface curvature, pinning effects, and the suspending conditions^5^.

Several fabrication methods based on lithographic techniques have been proposed to produce re-entrant textures^6^. Brown and Bhushan suggested that spherical and/or cylindrical curvatures are able to support high droplet contact angles^7^. Ellinas *et al*. assembled a dual-scale texture by spin coating and etching on the surface of poly(methyl methacrylate)^8^. Zhao *et al*. created a wavy structure at the top of pillars using photolithography as a model surface of re-entrant textures^9^. An overhang structure using reverse nanoimprint lithography was used in conjunction with reactive ion etching as a re-entrant texture^10^. Mushroom-like microstructures were fabricated by molding poly(dimethylsiloxane) onto a patterned sacrificial photoresist bilayer^11^. Another approach used the self-assembly of colloidal monolayers as a partial mask^12^. A fabrication process using laser ablation and electrodeposition was also investigated^13^. Wu *et al*. fabricated a microstructure with uniaxial cylindrical curvature on stainless steel using a femtosecond laser^14^. However, uniaxial structure such as cylindrical curvature has a possibility to show an anisotropy in wettability. Domingues *et al*. found a critical limitation of microtextures comprised of pillars that undergo catastrophic wetting transitions in the presence of localized physical damage and defects^15^. An efficient strategy when using microfabrication methods to produce re-entrant textures is to establish not only a hydrophobic surface but also an oleophobic surface.

The food industry requires a method to create surfaces that remain clean and do not leave packaging residue for applications such as food bottles, food containers, and preservation bags. Especially, it is needed a technique to prevent the dirt by residue occurring to pour the foods. In this area, recognizing time-dependent behavior of liquid as deforming, sliding, or rolling across the surface is critical for preventing the fly and/or splash phenomenon, which soils the containers. A strategy inspired by *Nepenthes* pitcher plants is proposed to create slippery liquid infused porous surface^16^. However, there is a problem that the infused lubricating fluid contaminates with the foods. The purpose of the study was to propose the proof-of-concept of a facile fabrication method for manufacturing hydrophobic/oleophobic surfaces suitable for use in the food industry and that are especially effective for liquid foods such as soy sauce (a seasoning made from a fermented soybean paste) and canola oil (a vegetable oil produced from rapeseed). First, a microfabrication methodology for re-entrant textures was developed by using poly (tetrafluoroethylene) (PTFE) microbeads and an ultra-violet-cure (UV-cure) resin. The PTFE microbeads and UV-cure resin were spin coated onto glass substrates to produce a re-entrant texture. Optimum conditions for the diameter and pitch of the microbeads were identified for the microfabrication process of the re-entrant texturing. Next, we demonstrated both experimentally and theoretically how the microfabrication parameters affect liquid repellence.

## Materials and Methods

### Chemicals

A UV-cure resin (Di-Pentaerythritol Hexa Acrylate, M-405, ARONIX, 3,700–5,700/25 mPa·s/°C viscosity, Toagosei Co., Ltd., Tokyo, Japan) and a glass substrate (25 mm diameter, 0.15 mm thickness, 12-545-102, Thermo Fisher Scientific K.K., Tokyo, Japan) were used to produce the re-entrant textures. To possess PTFE microbeads with diameters from a few micro-meters to over a thousand micro-meters, three types of microbeads were used (3 μm diameter, Microdispers-3000, Poly sciences, Inc., Warrington, PA; 35 μm diameter, 468096, Merck KGaA, Darmstadt, Germany; and >40 μm diameter, 182478, Merck KGaA, Darmstadt, Germany). The microbeads with diameters exceeding 40 μm were sorted according to prescribed diameters by using fifteen types of sieves (wire pitch: 53–1,000 μm, JIS Z 8801, Tokyo Screen Co. Ltd., Tokyo, Japan). In this way, microbeads with seventeen different diameters ranging from 2.26 ± 1.01 to 1,353 ± 190 μm [mean ± standard deviation (SD), *n* = 40] were prepared. Soy sauce (Kikkoman Co., Noda, Japan) and canola oil (Nisshin OilliO Group, Ltd., Tokyo, Japan) were used to evaluate the hydrophobicity/oleophobicity of the sample surfaces. A PTFE flat plate was used to measure equilibrium contact angles of distilled water and liquid foods (No. 9000, NAFLON, 0.092 ± 0.016 μm of arithmetic average roughness, Nichias Corporation, Tokyo, Japan).

### Microfabrication of re-entrant texture

**Figure 1:**
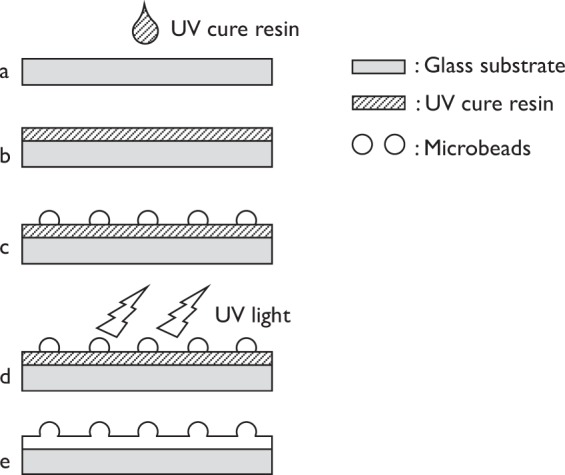
Fabrication process of re-entrant texture: (**a**) glass, (**b**) UV curing resin, (**c**) distribution of micro-beads, (**d**) UV irradiation, and (**e**) final re-entrant texture.

The re-entrant texturing was fabricated as follows (Fig. 1):

Step a: Use a brush to apply the UV-cure resin to the glass substrate.

Step b: Use a spin coater (ACT-220DII, Active Co. Ltd., Saitama, Japan) to regulate the thickness of the UV-cure resin.

Step c: Scatter a mass of microbeads of each diameter on the layer of UV-cure resin.

Step d: Irradiate the UV-cure resin with UV radiation from a UV lamp (FL4BLB, 0.2 W, 352 nm, 2.16 μW/cm^2^, Toshiba Lighting & Technology Co., Yokosuka, Japan).

Step e: This completes the microfabrication of a sample plate (sample with a spherical curvature).

### Tuning thickness of UV-cure resin

The thickness of the UV-cure resin was varied by controlling the rotation speed of the spin coater as a parameter ranging from 1,000 to 8,000 rpm. To measure the thickness of the UV-cure resin, the surface was scratched with a utility knife and the side view was observed using a noncontact laser confocal microscope (with a depth resolution of 1 nm, OLS4100, Olympus Co., Japan).

### Measurement of re-entrant texture

A side view of the sample plate verified the formation of the re-entrant texture. Two sample plates with medium-sized microbeads (diameters of 278 ± 48 and 323 ± 51 μm) were used for the evaluation (50 mg of microbeads).

Next, the relationship between microbeads pitch and sample plate rotation speed was measured using the mass of microbeads as a variable ranging from 10 to 50 mg (diameters of 278 ± 48 μm). The pitch was analyzed using the noncontact laser confocal microscope run by native software.

### Measurement of apparent contact angle and sliding angle

The hydrophobicity/oleophobicity of the sample plate was evaluated using soy sauce and canola oil, with distilled water used as the control. The surface tension, γ_LV_, of the liquid foods was measured by using the pendant drop method^17^ and a commercial contact angle analyzer (DM-701, Kyowa Interface Science Co. Ltd., Japan).

Ten μL of each sample liquid were placed softly onto the sample plate (50 mg of microbeads with diameter of 278 ± 48 μm) to evaluate the adhesiveness using the commercial contact angle analyzer.

Next, the apparent contact angle, θ′, and the sliding angle, α, of the sample plate fabricated using microbeads with diameters between 2.26 ± 1.01 and 1353 ± 190 μm (50 mg of microbeads) were measured by using distilled water and the commercial contact angle analyzer at 24 °C and 55% relative humidity. Sliding was judged to have occurred if the distilled water droplet moved more than 20 mm from its original position. Distilled water (3 and 6 μL for the apparent contact angle and 10.5 μL for the sliding angle) was dropped from a microsyringe onto the surface of each sample plate. The measurement method of the sliding angle was modified because a droplet cannot be placed directly on the sample plate. Droplets were therefore released onto samples from a height of 10 mm^18,19^. The measurements were repeated five times using different areas.

Finally, the apparent contact angle and the sliding angle of sample plate for soy sauce and canola oil were measured.

## Results and Discussion

### Tuning thickness of UV-cure resin

The thickness (8.0–2.7 μm) of the UV-cure resin on the glass substrate decreased proportionally with increasing rotation speeds from 1,000 to 8,000 rpm. The thickness was stable at 4.0–4.2 μm for rotation speeds in the range 4,000–6,000 rpm, so 5,000 rpm was used for the fabrication.

### Measurement of re-entrant texture

**Figure 2:**
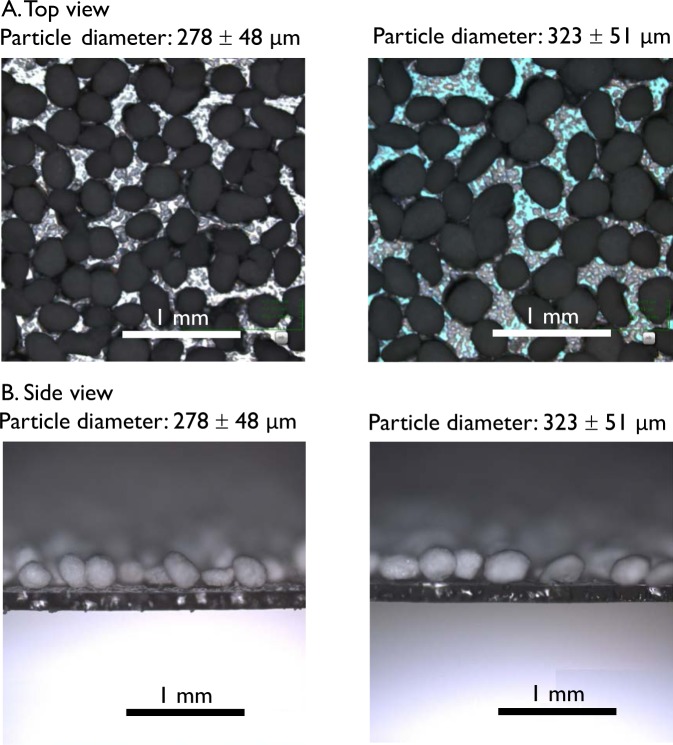
Top and side views of two samples with spherical curvature (50 mg of microbeads of diameters 278 ± 48 and 323 ± 51 μm).

**Figure 3:**
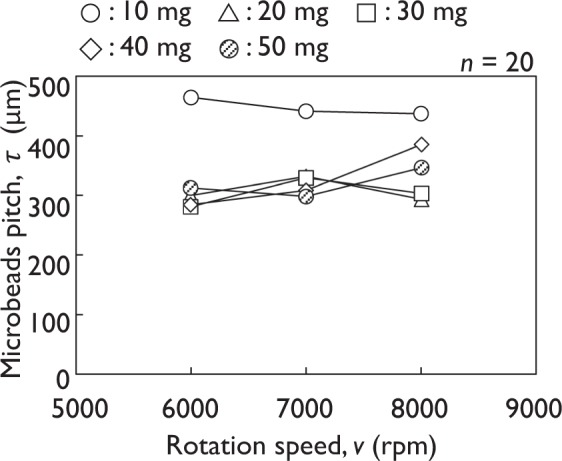
Relationship between microbeads pitch and spin coater rotation speed (microbead diameters: 278 ± 48 μm).

A side view of the sample plate revealed that a re-entrant texture, a spherical curvature, was formed (Fig. 2). The particle pitch of the sample with spherical curvature remained in a relatively narrow range of 282–386 μm between 6,000 and 8,000 rpm, except when using 10 mg of microbeads (*n* = 20, Fig. 3). The high rotation speed effectively removed the microbeads that did not adhere to the glass substrate. Thus, it was considered that 8,000 rpm and 50 mg of microbeads should be used to properly scatter the microbeads over the UV-cure resin. To scale up production, a method may be adapted to transfer the re-entrant texture to a replica-mold with a flexible material such as dimethylpolysiloxane and then transfer it to the final product.

**Figure 4:**
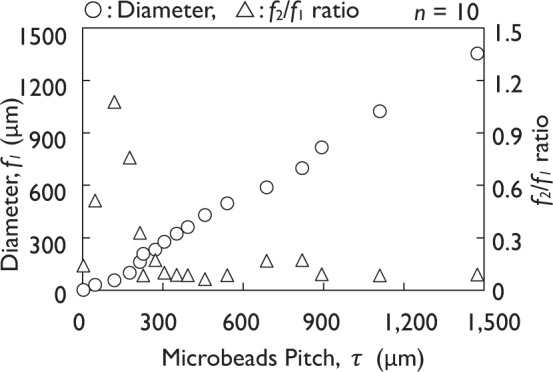
Relationship between geometric shapes of re-entrant textures.

The mean values were used for *f*_1_ and *f*_2_ because the microbead diameter and spacing between microbeads showed polydisperse characteristics caused by the fabrication method (*n* = 10, Fig. 4). The spacing between microbeads, *f*_2_, was small compared with the microbeads diameter, *f*_1_. As a result, the *f*_2_/*f*_1_ ratio ranged from 0.14 to 1.30.

### Measurement of apparent contact angle and sliding angle

**Figure 5:**
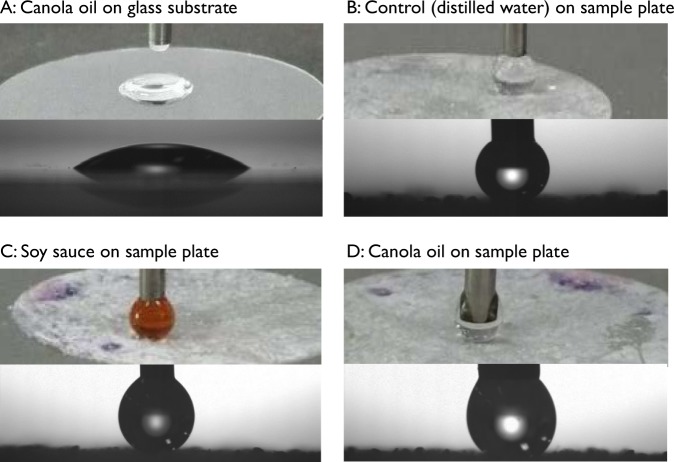
Hydrophobicity/oleophobicity of the sample with spherical curvature was evaluated by softly placing a droplet of distilled water, soy sauce and canola oil onto the sample surfaces (upper panels) and the goniometer images (lower panels, 50 mg microbeads with diameters of 278 ± 48 μm).

The adhesion of liquids on the sample with spherical curvature (microbeads with diameters of 278 ± 48 μm) was evaluated using distilled water, soy sauce, and canola oil. Each liquid was placed softly onto a given sample covered with spherically curved particles; however, the droplets could not be placed directly on the surface (Fig. 5).

**Figure 6:**
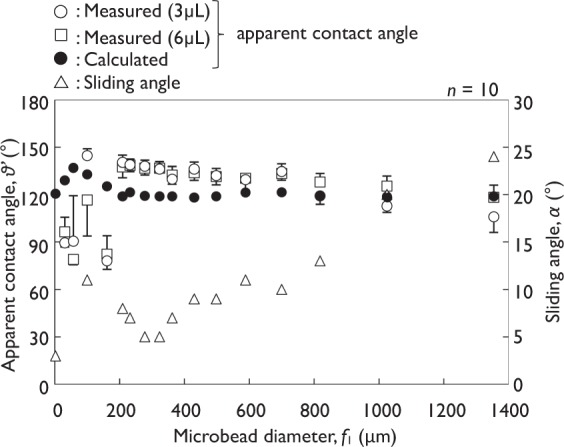
Apparent contact angle and sliding angle as a function of the microbead diameter of the sample (50 mg of microbeads, distilled water droplets, error bars are SD).

Upon placing by pushing a 3 μL droplet of distilled water (microbead diameters of 2.58–1,353 μm; *n* = 10; Fig. 6) onto the sample surfaces, the apparent contact angle, θ’, ranged from 73.7 to 144.6°. The use of 6 μL droplets gave a similar result of θ′ = 78.9–138.5°. A large different of the apparent contact angle was not observed between 3 and 6 μL droplets. For 3 μL droplets of distilled water, the maximum apparent contact angle of 144.6° occurred with microbead diameters of 100 ± 20 μm when 3 μL distilled water was used. The minimum sliding angle, α, for distilled water on surfaces with microperiodic structures was 5° when using microbead with diameters ranging from 278 ± 48 to 323 ± 51 μm (two samples).

**Figure 7:**
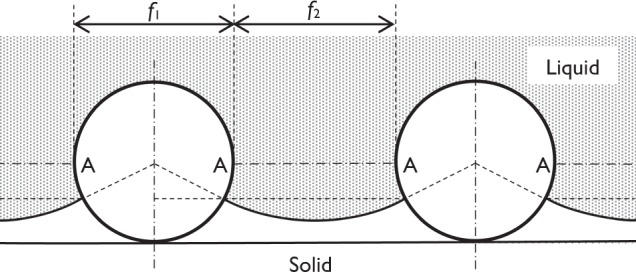
Schematic of a solid surface of re-entrant texture with a cylindrical model (AA: water position on cylinders under zero hydrostatic pressure).

The apparent contact angles, θ′, were calculated based on Cassie-Baxter equation of re-entrant texture with a cylindrical model (Fig. 7)^20,21^.

1 $$\cos \,{\theta }^{{\rm{^{\prime} }}}=-\,1+\frac{1}{{D}^{\ast }}\{(\pi -\theta )\cos \,\theta +\,\sin \,\theta \}$$

where, D* = (*f*_1_ + *f*_2_)/*f*_1_ = τ/*f*_1_,

*f*_1_: microbead diameter,

*f*_2_: spacing between microbeads (=τ − *f*_1_),

θ: equilibrium contact angle, θ of distilled water for PTFE = 108.4 ± 2.18° (*n* = 10)^22,23^.

This theoretical model adapts a hypothesis that the water is under zero hydrostatic pressure.

The measured apparent contact angle increased as the microbead diameter increases from 2.26 ± 1.01 to 100 ± 20 μm, and then gradually decreased as the microbead diameter increases further. Although there is a limitation that the geometric parameters of the surfaces distributed stochastically, the calculated results were consistent with the measured results (Fig. 6). These results showed that the water did not reach to the bottom of texture by suspended with the fabricated textures with spherical curvature in these experimental conditions. In addition, these results confirmed the validity of the theoretical equations proposed by Tuteja *et al*.

**Figure 8:**
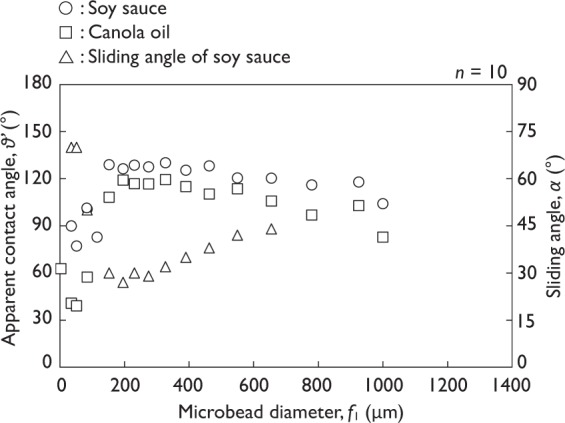
Apparent contact angle and sliding angle of soy sauce and canola oil (50 mg of microbeads, error bars are SD).

Re-entrant textures are not necessary to achieve the Cassie-Baxter state when the equilibrium contact angle over 90°. However, repelling oils and other complex liquids are difficult than water. Thus the apparent contact angle and sliding angle for soy sauce and canola oil droplets were measured by placing a droplets of each onto the sample surfaces (Fig. 8). The measured results of equilibrium contact angles of soy sauce and canola oil for the PTFE flat plate were 88.7 ± 2.58° and 69.9 ± 1.32°, respectively (*n* = 10). The measurement of the apparent contact angle was difficult when the microbead diameter was 1,353 μm because these values largely varied depending on the position of droplets. The maximum apparent contact angle and minimum sliding angle for soy sauce were 130.2° (*f*_1_ = 361.9 μm) and 27° (*f*_1_ = 232.0 μm), respectively. The maximum apparent contact angle and minimum sliding angle for canola oil were 119.4° (*f*_1_ = 361.9 μm) and >70°, respectively. The sliding angles of canola oil showed >70° throughout the measurement. The surface tensions of soy sauce and canola oil were 42.2 and 32.1 mN/m, respectively. The apparent contact angle of distilled water, soy sauce, and canola oil was thus proportional to the surface tension, γ_LV_, of the given liquid. These results indicated that the re-entrant textures showed the hydrophobicity/oleophobicity for liquid foods.

The viscosities of milks, alcoholic drinks, vegetable oils, juices, and other sources were have been reported as between 1 and 10^3^ mPa·s (=cP)^24^. The viscosities of soy sauce and canola oil were consistent with previous reports ranging from 10^1^ to 10^2^ mPa·s^25^. In addition, the viscosities of soy sauce and canola oil do not depend on the velocity gradient^24,25^, so these are classified as Newtonian liquids. The proposed microfabrication methodology for re-entrant texturing could thus be used to improve the hydrophobicity/oleophobicity for Newtonian liquid foods.

## Conclusion

A facile fabrication method of re-entrant textures with spherical curvature was proposed demonstrating improvements in the hydrophobicity/oleophobicity of Newtonian liquid foods. The apparent contact angles of the sample with spherical curvature were investigated in detail both experimentally and theoretically. The liquid repellence might prove useful for preventing soiling by liquid foods. The proposed methodology outlined herein may be adapted for applications such as food bottles, food containers, and preservation bags that require self-cleaning without leaving packaging residue.

Additional improvements may be required for non-Newtonian liquid foods with viscosities exceeding 10^3^ mPa·s, such as ketchup, mayonnaise, syrup, and butter, because the pinning effects become dominant. To date, few publications discuss time dependent wetting based on the Cassie-Wenzel transition^26,27^ for the viscous fluids, so this issue is left for future work. Also, it is needed for future studies to investigate wetting transitions in new factors such as the dissolution of the entrapped gas into the liquid, effects of mechanical vibrations, and breakthrough pressures^28,29^.
